# Perceptions of Produce Recall Responsibility and Compliance of FSMA Produce Safety Rule Among Small- and Medium-Sized Produce Growers

**DOI:** 10.3390/foods15162930

**Published:** 2026-08-21

**Authors:** Elma Kontor-Manu, Yaohua Feng

**Affiliations:** Department of Food Science, Purdue University, West Lafayette, IN 47906, USA

**Keywords:** produce safety, outbreak responsibility, produce outbreak, produce growers, small- and medium-size farms, Produce Safety Rule, packing houses, foodborne illnesses

## Abstract

Ensuring the safety of produce is essential for protecting consumers and preventing foodborne illnesses. Regulatory frameworks, such as the Food Safety Modernization Act’s Produce Safety Rule (FSMA PSR), contribute to the decision-making process of produce growers in ensuring the production of safe foods. However, little research has explored the role of growers’ perceptions of responsibility for produce-associated outbreaks, and how these may influence their decisions. Using a cross-sectional online survey of 500 US small- or medium-sized produce growers, this study examined perceptions of stakeholder responsibility for two produce-related outbreaks involving jalapeños and a spinach–spring mix blend. The implications of the PSR exemptions were also investigated. Responsibility attribution differed among stakeholder groups for each produce commodity. Packing houses were identified as a common denominator responsible for both produce commodities. Medium-sized produce growers agreed that the PSR exemptions provide direct market access and reduced time commitment to exempted farms (*p* < 0.001). However, small-sized produce growers expressed concerns about the potential limitations of the exemptions for qualifying farms. The results provide insights to policymakers, educators, and industry stakeholders to incorporate programs that foster shared responsibility for produce safety across the food system. It also informs future evaluations of the implications of the PSR exemptions.

## 1. Introduction

Produce safety is an important priority within the food supply chain and public health. Over the past decade, an increase in reported cases of foodborne illnesses and product recalls have been linked to produce [1,2,3,4]. This issue remains a public health concern, as it contributes to illnesses and economic losses [5]. The Food Safety Modernization Act Produce Safety Rule (FSMA PSR) provides regulatory guidelines for produce growers as preventive measures to avoid foodborne illnesses [6]. PSR imposes compliance obligations that growers must follow when making food safety decisions. However, growers also must take into consideration regulatory requirements, inherent risk perceptions, and constraints, among other factors [7,8,9].

Small- and medium-sized farms comprise the majority of the farming sector in the United States [10,11]. The USDA defines a small farm as an operation with a gross cash flow income (GCFI) of less than $350,000, and a medium farm as an operation with a GCFI between $350,000 and $999,999 [12]. Most produce farms are small- or medium-sized, with an income of less than $500,000 [13]. Although their contribution to US produce production value is smaller than that of large farms [14], small- and medium-sized farms equally contribute to the produce safety system, and bear the burden of foodborne outbreaks and recalls. Furthermore, many of these produce growers, especially those operating small-sized farms, qualify for an exemption from the FSMA PSR [13], so they are not as strictly regulated as other farms, even though they have access to direct consumer sales through farmers’ markets, roadside stands, and on-farm sales.

Food safety decision-making is a complex process, and the approaches applied can be diverse and dependent on a number of factors. Expert systems, Bayesian networks, and multi-attribute decision analysis are examples of methods that have been applied in some studies to support food safety decisions in certain areas [15,16,17]. Other studies have looked at food safety decisions through a behavioral and psychological lens, because in reality, these decisions may not be based entirely on a systematic or direct cost-benefit approach. There may be certain behavioral and cognitive aspects that influence these decisions that need to be included in the process. For example, researchers have employed frameworks such as the Theory of Planned Behavior (TPB), Health Belief Model (HBM), and other methods to understand what influences food safety decisions or contributes to adapting certain food safety behaviors [18,19,20,21]. By applying TPB in their evaluation, for example, Nickell & Hinsz [22] found that employees from different departments in the same processing plant had different determinants that influenced their decisions for safe food handling practices. Food safety decisions were shaped by the operational roles and perceptions. Other studies among farmers [23] and vendors [24], industrial food processing workers [25], and consumers [26] have demonstrated that food safety compliance behaviors differ across stakeholder groups, supporting the premise that there is no one-size-fits-all when it comes to understanding the food safety decisions of stakeholders in the food supply chain. The variability in stakeholders’ perspectives highlights the importance of identifying or understanding the determinants that influence these decisions.

Past research has examined various determinants of on-farm food safety decision-making, such as compliance barriers, grower knowledge, awareness, and implementation initiatives [7,13,27,28]. Findings from these studies have shown that food safety decisions among produce growers are not only based on regulations or available resources, but also on risk perceptions and responsibilities. Despite the numerous outbreaks and recalls associated with produce, one feature that has not been explored is how growers attribute responsibility in the event of a foodborne outbreak or recall. The food supply chain involves multiple actors at different stages, and the responsibility for food safety is shared among these actors. The attributions of these growers, especially those that are small- to medium-sized, may reflect whether they perceive actions or preventive measures as appropriate or necessary, which also may shape their willingness to adopt certain food safety practices. Social scientists refer to this phenomenon as attribution theory, which explains how people make causal attributions that influence their decisions regarding a behavior [29]. In this context, produce growers’ attribution of responsibility for an outbreak may influence their food safety decisions, and how they act on requirements.

A few studies have examined individual stakeholders’ contexts in responsibility attribution of outbreaks or recalls. However, little is known about whether this attribution differs across produce implicated in these outbreaks. Produce commodities may differ in their production environment, handling practices, supply chain structures, and outbreak histories. These differences may influence perceptions of which stakeholder in the supply chain might have contributed to a possible outbreak. Therefore, examining different produce commodities could provide insights into whether attribution is a generalized orientation of food safety or dependent upon the specific commodity. Another perspective that has limited research is the comparison of outbreak scenarios among stakeholders. Produce growers and consumers are likely to process an outbreak event through different cognitive lenses. Consumers operate under information asymmetry and heightened risk perceptions, while produce growers have production and operational experience as well as knowledge of regulatory structures. Evaluating how these attributions diverge across scenario outbreaks is another element that could be investigated, especially considering that some farms are exempted from the FSMA PSR and others are not. Since FSMA PSR is the current standard for all produce growers, understanding growers’ perceptions may give some insights into its influence on food safety decision-making. Reports of challenges in complying with this regulation include the ambiguity and perceived complexity of the regulatory expectations [8,30,31]. Researchers have documented the impact of the FSMA PSR on small- and medium-sized farms [13,32,33], as well as on the decision-making trade-offs involved in implementing food safety measures on farms [33,34,35]; however, limited information is available on compliance dynamics from an operational perspective.

Two important gaps remain in understanding responsibility attribution for produce safety outbreaks: whether responsibility attribution varies across different commodities, and whether produce growers and consumers differ in their perceptions of responsibility. For this study we posed the following research questions: (1) Does attribution of responsibility among produce growers vary systematically among distinct commodities for a hypothetical outbreak involving a spinach and spring mix blend, and jalapeños? (2) How do responsibility attributions for produce safety outbreaks diverge among produce growers and consumers when given outbreak scenarios? (3) How do small- and medium-sized produce growers perceive the impact of the PSR on food safety compliance? Exploring these perceptions could help contribute to the nuances in understanding the food safety behaviors of small- and medium-sized produce growers to provide evidence to inform initiatives that better align with the realities of these growers.

## 2. Materials and Methods

### 2.1. Study Design

This online cross-sectional survey study was designed to assess produce growers’ attribution of responsibility to stakeholders regarding produce outbreaks related to two types of produce: jalapeños and a blend of spinach–spring mix. Additionally, the study explored their perceptions of on-farm food safety compliance and the FSMA PSR. This survey was part of a comprehensive study that examined various aspects of produce growers’ practices, but this article focuses only on the food safety aspect.

### 2.2. Participants’ Recruitment

To be eligible, participants had to meet the following requirements: (1) grow produce, (2) operate in the United States, and (3) the farm must have an annual GCFI of less than $350,000 for small-sized farms, and between $350,000 and $999,999 for medium-sized farms [12]. The participants were recruited using a convenience sampling approach by Qualtrics (Provo, UT, USA), through their data pool to reach produce growers across the United States. A total of 500 participants were recruited for the study. They were compensated by Qualtrics after completion of the survey.

### 2.3. Study Questions

The questions for this study were categorized into three sections. The first section provided a list of stakeholders on the food supply chain. Participants were asked to select from the list who they would blame in two hypothetical scenarios: a multistate outbreak of *Salmonella Saintpaul* infection linked to jalapeños, and a Shiga toxin-producing *Escherichia coli* O157:H7 infection linked to a spinach and spring mix blend. This question was adopted from another study to view responses from this target population [23]. The outbreak vignettes were constructed to hold a general outbreak context as consistently as possible while varying the implicated commodity. The second section explored on-farm food safety compliance and the factors that influence compliance decisions. Participants were asked to rate, on a scale from 1 to 5 (1 = strongly disagree, 5 = strongly agree), strategies that could motivate their compliance decisions. The third section focused on participants’ perceptions of the FSMA PSR, specifically the exemptions for qualifying farms [9]. Participants were asked to rate the benefits and disadvantages of being exempt from the FSMA PSR on a scale of 1 to 5 (1 = strongly disagree, 5 = strongly agree). Participants’ demographic information was also collected. The survey questions were developed based on the study objective and prior studies examining the responsibility for outbreaks [23], and the impacts of the Produce Safety Rule on small- and medium-sized produce growers [9]. Prior to full administration of the survey, the questions, including check all that apply (CATA) stakeholder itemized lists, were reviewed by a panel of experts that included a food safety extension specialist and agricultural economists. The reviewers assessed the survey instrument for clarity, relevance, and consistency between the commodity scenarios and other parameters. The questions were further pilot-tested with a few individuals from Purdue University to assess the questions’ readability, clarity and survey functionality.

### 2.4. Statistical Analysis

A Cochran’s Q-test was conducted to assess whether there were any significant differences in the “check all that apply” responses of stakeholders responsible for an outbreak across the farm sizes. Construct validity was confirmed post hoc using the Cochran’s test (*p* < 0.01) to demonstrate that the CATA stakeholder list varied significantly by scenario condition rather than reflecting random selections. During the data analysis, the responses from a previous consumer study (*N* = 914; [23]) that asked participants the same question regarding only the spinach and spring mix blend were compared with the responses from this current study. Chi-square tests of independence were used to examine differences in attribution of responsibility for an outbreak between farm and consumer study groups. Frequency analysis was conducted on behavioral determinants influencing on-farm food safety compliance. Additionally, a logistic regression was used to assess the differences in the influence of these determinants based on farm size. Descriptive analysis (frequencies and percentages) was used to analyze responses regarding the perceptions of the FSMA PSR. A chi-square test was conducted to compare the distribution of the demographic characteristics for the impact of FSMA PSR between small- and medium-sized produce growers in the study. The differences in the Likert scale responses between the grower groups were assessed using the Mann–Whitney U test. The data extracted for this analysis was cleaned using Microsoft Excel 2021 spreadsheet (Microsoft Office LTSC Professional Plus, Redmond, WA, USA) prior to further analysis. All other statistical analyses were conducted in SAS Studio 9.04.01M8P022223 (SAS Institute Inc., Cary, NC, USA, 2026), with statistical differences observed at *p* ≤ 0.05.

## 3. Results

### 3.1. Participants’ Profile

A total of 500 individuals representing small- (*n* = 261) and medium-sized (*n* = 239) produce growers were included in the study. Statistically significant differences were observed between the two farm sizes for all demographic variables: the produce growers’ age, years in farming, educational level, combined total household income, and household location. Table 1 shows the distribution of the participants’ demographics. Most respondents were between 35 and 44 years old, accounting for 49.0% and 44.8% of the small- and medium-sized produce grower groups, respectively (*p* = 0.0008). Participants also differed significantly in farming experience (*p* < 0.0001). Among small-sized produce growers, 50.6% reported 5–15 years of experience, while medium-sized produce growers reported 16–25 years (53.6%) or more than 25 years (18.8%) of experience, compared to their small farm counterparts (39.9% and 9.6%, respectively).

### 3.2. Perceptions of Stakeholder Responsibility for Produce Outbreaks

**Figure 1:**
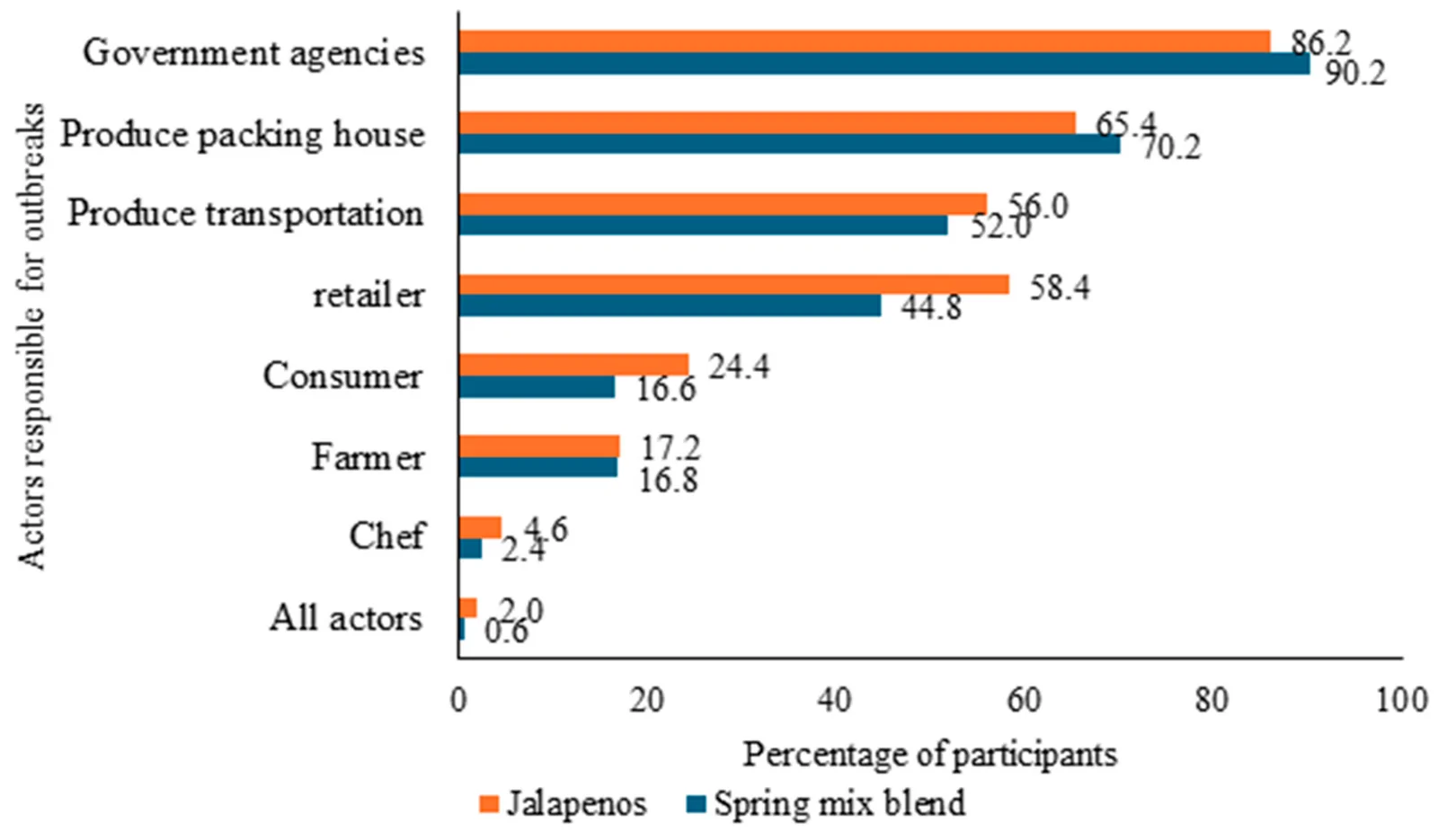
Percentages of selected actors responsible for foodborne outbreaks related to specific produce by all study participants (N = 500).

Participants were asked to indicate which stakeholder in the produce supply chain was responsible for an outbreak related to two specific crops: jalapeños, and spinach and spring mix blend. Figure 1 shows the overall response for both farm categories. The majority of the participants identified government agencies as most responsible for an outbreak involving jalapeños (86.2%) and the spinach–spring mix blend (90.2%), followed by produce packing house (65.4% for jalapeños, and 70.2% for spinach–spring mix blend), and produce transportation (56.0% and 52.0%, respectively). Only a few of the participants selected farmers (17.2% and 16.8%, respectively), and chefs (4.6% and 2.4%) as most responsible for an outbreak involving the two produce types.

Further statistical analysis showed significant differences in responses between the small- and medium-sized produce growers. When asked who should be blamed more in an outbreak related to jalapeños, a Cochran’s Q test indicated that growers had statistically significant responsibility for the outbreaks across the different stakeholder categories for both small- and medium-sized produce growers (a Cochran’s Q test, small farm: Q = 655.59, *p* < 0.0001; medium farm: Q = 678.68, *p* < 0.000). The same outcomes were seen with the spinach and spring mix blend (small-sized produce growers: Q = 716.83, *p* < 0.0001; medium-sized farm: Q = 792.45, *p* < 0.000). This suggests that growers were not randomly selecting actors, but rather they consistently blamed certain stakeholders more than others.

Following up with chi-square tests, the results showed that for the spinach and spring mix blend, medium-sized produce growers were more likely to identify produce transportation as responsible for an outbreak (57.3% versus 47.1%, *p* = 0.029), while small-sized produce growers identified retailers (49.8% versus 39.3%, *p* = 0.024) and consumers (20.3% versus 12.6%, *p* = 0.027) as most responsible. For jalapeños, the only statistical difference observed was that small-sized growers were more likely to select consumers than medium-sized growers (28.4% versus 20.1%, *p* = 0.041). Table 2 shows the results from the chi-square tests.

**Figure 2:**
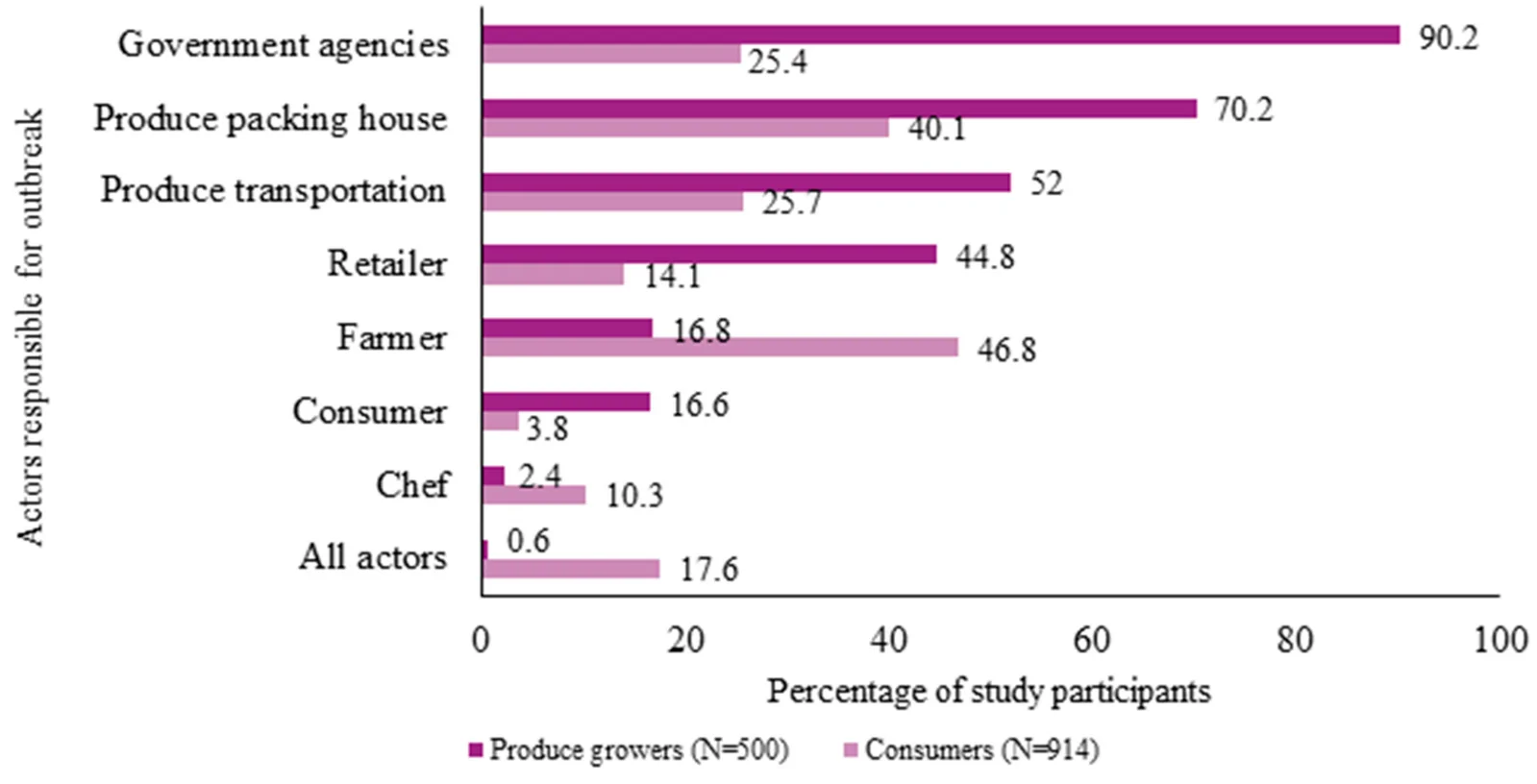
Comparison of responses between produce growers and consumers for an outbreak related to a spinach and spring mix blend.

The responses from this current study were compared with those from a previous study [23], which explored the same parameter of stakeholders responsible for an outbreak related to a spinach and spring mix blend among consumers (Figure 2). Findings indicate that both groups (produce growers and consumers) perceive stakeholder influence quite differently for an outbreak related to a spinach and spring mix blend (all responses: *p* < 0.0001). Produce growers largely selected government agencies as responsible for the outbreak rather than the consumer group (90.2% vs. 25.4%, *p* < 0.0001). They also were more likely to select the produce packing house (70.2% versus 40.1%, *p* < 0.0001), produce transportation (52.0% versus 25.7%, *p* < 0.0001), and retailers (44.8% versus 14.1.%, *p* < 0.0001). Interestingly, the consumer group more often selected farmers as responsible (46.8% versus 16.8%, *p* < 0.0001). This finding illustrates a clear distinction in perceptions of where responsibility lies within the produce supply chain for both growers and consumers for an outbreak related to a spinach and spring mix blend (Table 3).

### 3.3. On-Farm Food Safety Compliance

**Figure 3:**
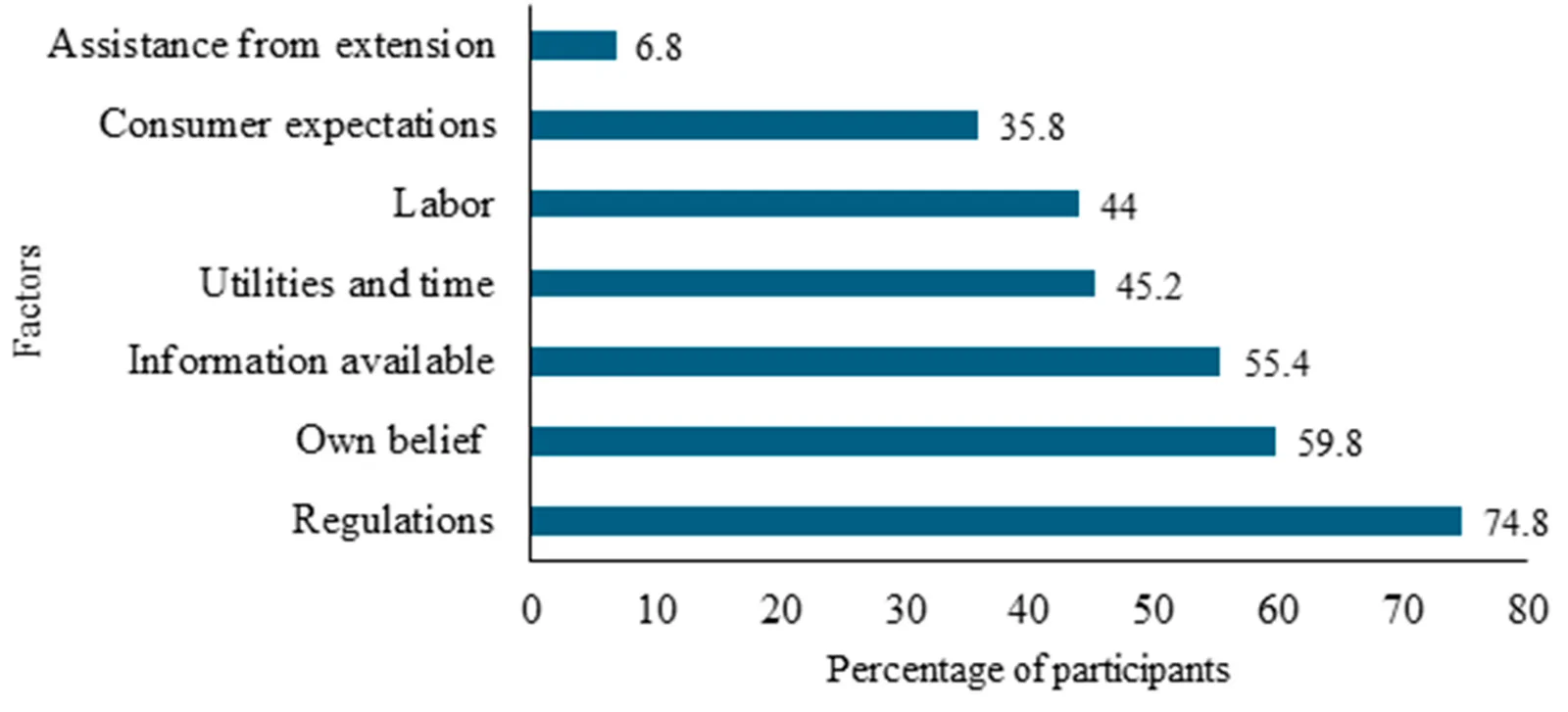
Various determinants that contribute to food safety decisions.

Multiple determinants were explored to identify what influenced produce growers’ food safety decisions. Overall, the most selected determinants with more than half of the general participants’ population were regulations (74.8%), personal/own beliefs (59.8%), and information available (55.4%), as seen in Figure 3.

Logistic regression examined whether behavioral determinants were influenced by small- or medium-sized produce growers. Medium-sized produce growers were significantly more likely to report that their food safety decisions were driven by their own beliefs (OR = 1.605, *p* = 0.0104) while less likely to be driven by regulations (OR = 0.555, *p* = 0.0048) (Table 4). This suggests that medium-sized growers were influenced more by their own beliefs, while small-sized growers were more influenced by regulations.

Growers’ perceptions of FSMA PSR exemptions were also investigated, with notable differences between small- and medium-sized produce growers (Table 5). Significant differences were found in responses on exemptions of FSMA, based on farm size, age group, farming experience, and household income. Small-sized produce growers were more likely to answer in the affirmative to farms being exempted from the rule (42.5%) compared to medium-sized farms (5.9%, *p* < 0.0001). Farmers with 26 years or more farming experience were predominantly against the exemption of certain farms from the rule (90.0%, *p* < 0.0001). Farmers with relatively fewer years in farming were more likely to respond “Yes” in support of small farms being exempt from the FSMA PSR.

### 3.4. Benefits and Limitations of PSR Exemptions

Perceptions of the benefits of the PSR differed between small- and medium-sized produce growers. A statistically significant difference was observed, with medium-sized growers expressing higher levels of agreement than small-sized growers with statements regarding the benefits of exemption in promoting direct market sales (*p* < 0.0001), and reducing time commitment (*p* < 0.0001). Both groups recognize the benefits, but medium-sized farms appear to be in more agreement with the beneficial impact of PSR exemptions (Table 6).

A statistically significant difference was observed between the two farm groups for perceptions related to the limitation of the FSMA PSR exemptions. Small-sized produce growers had significantly higher mean ranks for all statements, indicating higher levels of agreement, than medium-sized produce growers (Table 7). Small-sized growers expressed greater concern about reduced profits, with higher agreement levels, compared to medium-sized growers, who were more likely to disagree, suggesting greater financial resilience or confidence in sustaining profits. A similar outcome was seen with the impact on growth in farm business expansion. Medium-sized produce growers were more likely to disagree compared to small-sized produce growers, with 54% totaling in disagreeing and strongly disagreeing (Figure S2). 

## 4. Discussions

This study examined constructs of food safety decisions made by small- and medium-sized produce growers across the United States, with a focus on their perceptions of responsibility for illness outbreaks and recalls, and the effectiveness of FSMA’s Produce Safety Rule. Findings show that these growers perceive other stakeholders outside the farm level as responsible for an outbreak related to jalapeños, and a spinach and spring mix blend. Growers’ perceptions of the FSMA PSR also revealed their views on exemptions from the rule, as well as its benefits and disadvantages.

### 4.1. Responsibility Attribution and Food Safety Decision-Making Between Small- and Medium-Sized Produce Growers

The findings indicated that both small- and medium-sized produce growers attributed responsibility for jalapeño, and spinach and spring mix contamination to other stakeholders, particularly government agencies, produce packhouses, and transportation (distributors). This reflects one of the fundamental constructs of attribution theory, which stipulates that when an event occurs, individuals evaluate the cause under different dimensions, with the locus of the cause being one of these dimensions. The cause could be internal, which is a personal causality or within the individual’s direct control, or external to the individual [36]. Both produce grower groups assigning responsibility for these outbreaks predominantly to other groups indicates that growers externalize the locus of cause, which suggests that on-farm food safety protocols were maintained, thereby detaching farm-level operations from post-harvest events. Another notable finding of this study was that the majority of participants attributed these hypothetical outbreaks to government agencies. This reallocation of responsibility aligns with another dimension of attribution theory, which is controllability. This dimension suggests that responsibility for a cause can be explained if the individual or actor in question could have controlled or changed the cause [36] (e.g., stakeholders with greater capacity to mitigate the outbreak event). Government agencies or regulators play several roles within the food safety system, including establishing and enforcing standards, conducting traceback investigations and inspections across all areas of the food supply chain [37,38]. The selection of government agencies may reflect growers’ perceptions of the role these agencies play in one or more of the oversight functions, rather than beliefs that government agencies directly caused the outbreak. Aligning with the multi-country study findings reported by Pinho-Gomes and colleagues [39], participants assigned the responsibility of food policy and regulations to the government, highlighting how structural environments shape judgements of causal controllability.

### 4.2. Commodity-Specific Heterogeneity in Responsibility Attribution

The findings from this study further demonstrate that produce growers’ evaluation of responsibility attribution varied across the distinct produce commodities. Small- and medium-sized produce growers indicated potential responsibility attribution for all three stakeholders (consumers, produce transporters, and retailers) in the spinach and spring mix blend scenario, while responsibility was observed in only one stakeholder group (consumers) for jalapeños. This may be explained by the differences in production systems, contamination pathways, and perceived stakeholder control between the two produce commodities. The spinach and spring mix blend supply chain is complex, involving multiple actors and facing heavy regulatory scrutiny as a leafy green product [40,41,42]. The multi-ingredient supply chain of this commodity, which ranges from harvesting and washing to packing and distribution, introduces multiple transportation and distribution points [43,44], creating structural opacity. Consequently, growers may differ in their perceptions of where responsibility lies within this system. On the other hand, jalapeños are a one-item commodity, and their production-to-distribution chain may be shorter, involving fewer multiple-tier handoffs [45].

### 4.3. Divergence in Stakeholder Responsibility Attribution

In contrast, produce growers and consumers had clearly different views on who was responsible for an outbreak involving a spinach and spring mix blend. Consumers predominantly attributed the event of an outbreak to produce growers. This observation could be consistent with the concept of information asymmetry, which refers to the imbalance of information accessibility and use among stakeholders [46,47]. The complexity of the supply chain creates a system in which information asymmetry is common. For example, one stakeholder group may have more information on a subject than another, or both groups may have varying information levels on the same subject [48,49]. Gómez-Aguaz shares the disparity in information availability among the different stakeholder groups in the agri-food industry, emphasizing a difference in the measure of food safety and quality [50]. The findings from this study suggest that consumers may not be fully exposed to the complexities of the food supply chain. As a result, they may not recognize the multiple points of possible contamination across the food supply chain. They may instead rely on available heuristics, such as publicly available information or media reports, to make judgments, which has become a common pattern in decision-making among consumers [51,52]. As end users of the product, consumers may perceive farms as the first point of contact, and expect produce safety to be managed at that stage, before it enters the marketplace [23,53]. The current study aligns with findings reported by Larson [54], who examined consumers’ perceptions of food safety responsibility in multiple countries, including the US. Those findings indicated that consumers were the least responsible for food safety among other supply chain members. Even though there were varied responses for other stakeholders in that study, farmers and retailers were implied to be more responsible for food safety. Another study with similar findings was conducted by Erdem and colleagues [55]. They reported that consumers found farmers to be more responsible for ensuring meat safety, while farmers perceived consumers to be more responsible. Growers’ different perspectives on responsibility may be due to their greater awareness of the dynamic and complex food system and the multiple possible points of contamination. These differences in responses may also reflect the disconnect between public expectations and operational realities [56,57].

### 4.4. Perceptions of FSMA Produce Safety Rule Exemptions

The perceptions of growers about the FSMA PSR illustrate the role regulations play in food safety decision-making and compliance. Findings from this study indicate differences in the growers’ responses with respect to the exemptions of the PSR for qualifying farms. A higher proportion of medium-sized produce growers disagreed with these exemptions, compared to those of small-sized produce growers. The preferences of small-sized produce growers for the exemption are consistent with the findings of studies that have reported on the financial constraints and the burden of the regulations, and how they are not well-tailored to small operations [28,58,59]. Enderton et al. [33] noted that even after years of being trained and implementing the PSR, growers still reported the challenge of time and financial constraints affecting their ability to make robust changes to expected infrastructure, such as handwashing stations in farm packhouses. These are important factors in growers’ decision-making process. For example, even if a grower knew what was required by the rule and wanted to comply, certain constraints would limit their ability to do so, which highlights the importance of understanding the role this construct may play in their decision-making.

Furthermore, from a decision-making standpoint, the FSMA PSR may serve as an overarching structure for growers’ decisions. It is important to note that the FSMA PSR may have a dual effect on growers, influencing their perspectives and, consequently, their decisions. This is made clear by Bovay & Sumner [60], who reported the probable effect of the FSMA PSR on different produce growers. They mentioned that small farms that do not qualify for exemption will be disadvantaged relative to other farms, and farms that have adopted other regulatory standards similar to FSMA would be at an advantage. This demonstrates that the rule not only provides science-based preventive measures but may redefine the economic context for food safety decisions [61]. The same goes for qualifying exemptions. For some growers, the rule may provide clarity and legitimize important food safety practices for reducing contamination at the farm level. The rule may benefit farms that already engage in third-party certifications as well as those that are exempt. On the other hand, for growers who are not exempt, such as small-sized produce growers, the cost of the regulation may influence their decisions regarding food safety priorities. The perceptions of the FSMA PSR’s benefits and disadvantages, as well as its exemptions, may reflect economic feasibility, which shapes food safety decision-making.

This study included responses from certain groups of participants who disagreed with the exemptions for qualifying farms. Farmers who were older and had more farming experience did not support exempting qualifying farms from the PSR. This could be because their experiences with produce contamination events led them to believe that the rule is a benchmark for all growers to navigate such events. Duong et al. [62] shared that socioeconomic factors, such as farm size and farming experience, had an effect on how farmers perceive and adopt risk management strategies. For instance, farmers with large farms were more concerned with production risks and therefore likely to adopt management strategies. Because the FSMA PSR is a risk-based preventive measure, small- and medium-sized produce growers may have different perspectives that could ultimately shape their behavioral choices.

### 4.5. Benefits and Limitations of FSMA Produce Safety Rule Exemptions

The findings highlight that growers’ perceptions of the benefits and limitations of the PSR exemptions can be influenced by farm size. Medium-sized produce growers were more likely to agree that the exemptions supported operational opportunities in terms of time commitment and marketing opportunities in terms of direct market access. This may be because, as medium-sized farms, they are more likely to be non-exempt and therefore more familiar with the benefits of direct market contact and the limited time involved in ensuring compliance with the rule in daily activities. It is well documented that one of the challenges with regulations such as the PSR is the time involved in carrying out some of the activities [7,9]. Enderton and colleagues reported that limited time was one primary barrier that prevented growers from executing more operational changes [33]. Such familiarity may have been a contributing factor in their perception of this as a positive development for exempted farms. Most medium-sized produce growers use intermediary marketing channels, which fall between direct and commodity channels, such as supermarkets and distributors, to sell their products [63,64]. These might give them the knowledge of the potential advantages of direct marketing for farms that are able to capitalize on direct consumer markets for sales. In addition, even if a medium-sized farm is exempt from the PSR, access to these intermediary channels requires full regulatory compliance and audits [13,65], limiting their regulatory flexibility and further highlighting the advantage of a direct market.

In contrast, small-sized produce growers were more in favor of the limitations that PSR exemptions presented. The findings suggest that these growers may emphasize the potential business risks associated with exemption status, as highlighted by their agreement with regard to potential liability issues, reduced profits, restricted business growth, and limited market reach. This finding shows that regulatory exemption does not equate to market exemption. While farms that are exempt may benefit from being exempt from legal requirements, this can lead to isolation from more markets, which may subsequently affect potential business and economic gains. For example, as previously mentioned, larger market channels usually require certain certifications and audits, which can limit farms that are exempt, as some of these certifications may require practices outlined in the rule [13,32,66]. It has been reported that small farms have limited financial capacities and narrower profit margins [61]. Therefore, such audits may be unfeasible for some small farms [67]. Collectively, the findings suggest that the perceived value of PSR exemptions depends on how growers assess trade-offs, such as market opportunities, and business or financial risks.

### 4.6. Practical Implications

These findings have important implications for food policymakers, educators, and other industry stakeholders seeking to strengthen food safety initiatives. This study reinforces the concept of a shared responsibility framework among stakeholders across the food supply chain [68,69,70]. This was highlighted by the emergence of divergent responses on responsibility attribution in an outbreak event, suggesting that growers are cognizant of the fact that outbreak events can result from oversights at different points in the supply chain. This underscores the interconnection that characterizes the supply chain, and indicates value in emphasizing shared responsibility across the farm-to-fork continuum. Extension and outreach programs could leverage this in programs by linking farm-level food safety practices to food safety outcomes in the downstream supply chain. Programs or public messaging could incorporate system-based case studies to demonstrate how contamination may occur at multiple points throughout the supply chain, while emphasizing preventive actions by each stakeholder. In addition, information alignment among stakeholders could contribute greatly to bridging the gap with regard to produce handling from upstream to downstream in the supply chain. The observations on PSR exemptions also suggest opportunities for Extension and other interested stakeholders to provide guidance on the purpose, scope, and clarity of the PSR exemptions.

## 5. Limitations

This study’s protocol was carefully designed to answer the research objectives. It focused on the responsibility of attribution related to a hypothetical scenario of an outbreak related to specific commodities: jalapeños, spinach and spring mix blend. Hypothetical scenario approaches can be good for examining perceptions; however, they may present some biases that may result in differences in responses during real-world scenarios [71]. Factors such as participants’ existing and current understanding of previous food safety incidents involving the commodity, regional differences, and regulatory experiences may play a part in judgments. This study did not investigate these factors, and future studies could investigate the role in which such factors may influence responses. In addition, even though this scenario design allows researchers to compare participants’ responses, they cannot reproduce the emotional, economic, or operational pressures associated with an actual outbreak. Produce growers directly involved in an outbreak may perceive responsibility differently when facing losses or investigations. Another limitation is that the sampling frame may underrepresent growers who have limited internet access, limited English proficiency, or do not participate in online research panels. The sample size for produce growers was not designed to reproduce the national distribution of produce growers by region or commodity. The findings of this study provide evidence of patterns and associations within the surveyed sample; however, caution should be exercised when generalizing these findings to a broader study population of US small- and medium-sized produce growers. Finally, although the questionnaire underwent expert review and pilot testing for clarity, relevance, readability, and functionality, the CATA stakeholder responsibility items were not subjected to formal psychometric validation or cognitive interviewing. Future research could further evaluate the validity and reliability of these measures across populations and outbreak contexts.

## 6. Conclusions

This study investigated the constructs of responsibility for produce outbreaks and perceptions of the FSMA Produce Safety Rule as they pertain to food safety decision-making among small- and medium-sized produce growers. It demonstrated the variances in perceptions of responsibility with respect to produce outbreaks. Examining stakeholder responses through the lens of attribution theory and farm scale dynamics revealed that responsibility attribution during a foodborne outbreak event could be conditioned by the commodity-processing complexity or information asymmetry. Addressing these may require operationalizing a shared responsibility framework across the supply chain. To build on these findings, future research could further explore the mechanisms underlying responsibility attribution for produce outbreaks among stakeholders through interviews or focus group discussions. Constructs that drive these perceptions can be further investigated to draw direct causative conclusions. Longitudinal studies could determine how growers’ attribution evolves following an actual commodity recall to track insights into the cognitive mechanism underlying long-term food safety adoption. Future research could also include large-scale operations to examine whether responsibility attribution and perceptions of food safety differ across farm sizes and production systems. The role of the FSMA Produce Safety Rule is another construct that was investigated, in particular, the exemptions for qualifying farms. The rule was highlighted as a compliance structure that may influence food safety decisions among small- and medium-sized produce growers, with exemptions viewed through the lens of potential market or business risk, depending on the farm size. Individuals’ cognition and compliance behaviors are not static; future studies could conduct longitudinal studies to track produce growers’ attitudes and attributional patterns over a period with updates and more exposure to the Produce Safety Rule.

## Figures and Tables

**Table 1:** Demographic characteristics of study participants (N = 500).

Characteristics	Small-Sized Produce Growers (N = 261)	Medium-Sized Produce Growers (N = 239)	*p*-Value
	% (n)		
Age			
25–34 years	21.1 (55)	11.3 (27)	0.0008 *
35–44 years	49.0 (128)	44.8 (107)	
45–54 years	25.7 (67)	33.9 (81)	
55–64 years	4.2 (11)	10.0 (24)	
Farming experience			
5–15 years	50.6 (132)	27.6 (66)	<0.0001 *
16–25 years	39.9 (104)	53.6 (128)	
26 years and above	9.6 (25)	18.8 (45)	
Education			
Did not complete high school	1.2 (3)	0(0)	0.0034 *
Grade 12 or GED	31.8 (83)	20.9 (50)	
College 1 to Year 3	49.0 (128)	56.1 (134)	
4-year college graduate	17.2 (45)	18.8 (45)	
Master’s degree	0.8 (2)	4.2 (10)	
Total household income			
$49,999 and less	24.9 (65)	0 (0)	<0.0001 *
$50,000–$74,999	33.0 (86)	2.1 (5)	
$75,000–$99,999	21.1 (55)	8.4 (20)	
$100,000–$149,999	13.0 (34)	30.1 (72)	
$150,000–$199,999	7.7 (20)	35.2 (84)	
$200,000 and above	0.4 (1)	24.3 (58)	
Residential location			
Urban	12.3 (32)	10.0 (24)	<0.0001 *
Suburban	67.1 (175)	49.0 (117)	
Rural	20.7 (54)	41.0 (98)	
Household located			
Rural area	6.1 (16)	16.3 (39)	<0.0001 *
In a town less than 2500	11.5 (30)	9.2 (22)	
In a town from 2500 to 100,00	42.2 (110)	49.0 (117)	
In a city from 10,000 to 50,000	30.7 (80)	20.1 (48)	
From 50,000 to 100,000	5.4 (14)	5.0 (12)	
In a city/urban area of 100,000 or more	4.2 (11)	0.4 (1)	

* Chi-square test was used to test significant differences among demographic groups for the farm sizes, with significance at *p* ≤ 0.05.

**Table 2:** Selected actors responsible for foodborne outbreaks based on farm size.

Actors	Spinach and Spring Mix Blend			Jalapeños		
	Small-Size (95% CI)	Medium-Size (95% CI)	*p*-Value	Small-Size (95% CI)	Medium-Size (95% CI)	*p*-Value
Chef	3.8 [1.5–6.1]	0.8 [0.0–2.0]	0.058	6.5 [3.5–9.5]	2.5 [0.5–4.5]	0.055
Consumer	20.3 [15.4–25.2]	12.6 [8.4–16.8]	0.027 *	28.4 [22.9–33.8]	20.1 [15.0–25.2]	0.041 *
Farmer	18.4 [13.7–23.1]	15.1 [10.5–19.6]	0.382	18.4 [13.7–23.1]	15.9 [11.3–20.5]	0.536
Government agencies	87.7 [83.1–91.4]	92.9 [89.6–96.2]	0.074	86.6 [82.5–90.7]	85.8 [81.3–90.2]	0.893
Produce packing house	70.1 [64.6–75.7]	70.3 [64.5–76.1]	1.000	64.8 [59.0–70.6]	66.1 [60.1–72.1]	0.822
Produce transportation	47.1 [41.1–53.2]	57.3 [51.2–63.6]	0.029 *	51.7 [45.7–57.8]	60.7 [54.5–66.9]	0.055
Retailer	49.8 [43.7–56.0]	39.3 [33.1–45.5]	0.024 *	59.8 [53.8–65.7]	56.9 [50.6–63.2]	0.576
All actors	0.8 [0.0–1.8]	0.4 [0.0–1.2]	1.000	2.3 [0.4–4.1]	1.7 [0.0–3.3]	0.754

* Chi-square test was used to test significant differences between farm sizes for each actor in the produce supply chain, with significance at *p* ≤ 0.05. CI is confidence interval.

**Table 3:** Actors responsible for an outbreak involving a spinach and spring mix blend: Produce growers versus consumers.

Actors	Produce Growers (95% CI) (N = 500)	Consumers (95% CI) (N = 914)	χ^2^
Chef	2.4 [1.1–3.7]	10.3 [8.3–12.2]	<0.0001 *
Consumer	16.6 [13.3–19.9]	3.8 [2.6–5.1]	<0.0001 *
Farmer	16.8 [13.5–20.1]	46.8 [43.6–50.1]	<0.0001 *
Government agencies	90.2 [87.6–92.8]	25.4 [22.6–28.2]	<0.0001 *
Produce packing house	70.2 [66.2–74.2]	40.1 [37.3–43.7]	<0.0001 *
Produce transportation	52.0 [47.6–56.4]	25.7 [22.9–28.5]	<0.0001 *
Retailer	44.8 [40.4–49.2]	14.1 [11.9–16.4]	<0.0001 *
All actors	0.6 [0.0–1.3]	17.6 [15.2–20.1]	<0.0001 *

* Chi-square test was used to determine significant differences between produce growers and consumers for each actor in the produce supply chain, with significance at *p* ≤ 0.05. CI is confidence interval.

**Table 4:** Logistic regression estimates of determinants by participants’ farm size (reference = small-sized produce growers).

Decisions	β-Estimate	OR Estimate	95% CI	*p*-Value
Own belief	0.4728	1.605	[1.118, 2.303]	0.0104 *
Information available	0.0193	1.019	[0.716, 1.451]	0.9148
Labor	0.1900	1.209	[0.849, 1.722]	0.2924
Utilities and time	0.0315	1.032	[0.725, 1.468]	0.8612
Regulations	−0.5891	0.555	[0.369, 0.835]	0.0048 *
Consumer expectations	−0.2999	0.741	[0.513, 1.071]	0.1103
Assistance from extension	0.2209	1.247	[0.621, 2.505]	0.5348

* Significant differences for binary logistic regression estimating behavioral determinants, reported at *p* ≤ 0.05.

**Table 5:** Participant responses on whether small farms should be exempted from the PSR.

Factors	No	Maybe	Yes	*p*-Value
Farm size				
Small	45.2 (118)	12.3 (32)	42.5 (111)	<0.0001 *
Medium	80.7 (193)	13.4 (32)	5.9 (14)	
Age				
25–34 years	48.8 (40)	23.2 (19)	28.0 (23)	<0.0001 *
35–44 years	55.7 (131)	12.3 (29)	31.9 (75)	
45–54 years	73.0 (108)	10.1 (15)	16.9 (25)	
55–64 years	91.4 (32)	2.9 (1)	5.7 (2)	
Farming experience				
5–15 years	46.0 (91)	15.6 (31)	38.4 (76)	<0.0001 *
16–25 years	67.7 (157)	12.5 (29)	19.8 (46)	
26 years and above	90.0 (63)	5.7 (4)	4.3 (3)	
Household income				
$49,999 and below	76.9 (50)	9.2 (6)	13.8 (9)	<0.0001 *
$50,000–$74,999	46.2 (42)	15.4 (14)	38.5 (35)	
$75,000–$99,999	49.3 (37)	13.3 (10)	37.3 (28)	
$100,000–$149,999	51.9 (55)	20.8 (22)	27.4 (29)	
$150,000–$199,999	74.0 (77)	6.7 (7)	19.2 (20)	
$200,000 and above	84.7 (50)	8.5 (5)	6.8 (4)	
Residential location				
Rural	69.6 (39)	5.4 (3)	25.0 (14)	0.0832
Suburban	59.6 (174)	12.3 (36)	28.1 (82)	
Urban	64.5 (98)	16.4 (25)	19.1 (29)	

* Chi-square test was used to determine significant differences for responses on PSR exemption, with significance at *p* ≤ 0.05.

**Table 6:** Differences in responses on the benefits of FSMA PSR exemptions among small- and medium-sized growers using the Mann–Whitney U test.

Benefit	Small (n = 261)	Medium (n = 239)		
	Mean Rank Scores		Z	*p*-Value
Promotes direct sales	226.31	276.92	4.17	<0.0001 *
Less inspections	247.43	253.85	0.51	0.6095
Small farm recognition	245.15	256.34	0.90	0.3675
Reduced time commitment	221.57	282.10	4.83	<0.0001 *
Reduced financial burden	241.13	260.74	1.58	0.1139

* Mann–Whitney U test was used to determine significant differences between growers’ responses, with significance at *p* ≤ 0.05.

**Table 7:** Differences in responses on the limitations of FSMA PSR exemptions among small- and medium-sized growers using the Mann–Whitney U test.

Limitations	Small (n = 261)	Medium (n = 239)		
	Mean Rank Scores		Z	*p*-Value
Liability issues	263.63	236.16	−2.31	0.0214 *
Reduced profits	282.76	215.27	−5.34	<0.0001 *
Reduced growth in farm business expansion	287.35	210.26	−6.21	<0.0001 *
Discourages market reach	275.56	223.13	−4.31	<0.0001 *

* Mann–Whitney U test was used to determine significant differences between growers’ responses, with significance at *p* ≤ 0.05.

## Data Availability

The data presented in this study are available on request from thecorresponding author. The reason is that the human subject data are not supposed to be published without restrictions.

## Supplementary Materials

The following supporting information can be downloaded at https://www.mdpi.com/article/10.3390/foods15162930/s1, Figure S1: Distribution of foods produced by small- and medium-sized produce growers (N = 500); Figure S2: Produce growers’ perspectives on the benefits of PSR exemptions: A Likert-scale analysis (N = 500); Figure S3: Produce growers’ perspectives on the limitations of PSR exemptions: A Likert-scale analysis (N = 500); Figure S4: Frequency of responses to benefits of PSR exemptions by farm size (small vs. medium-sized produce growers); Figure S5: Frequency of responses to limitation of PSR exemptions by farm size (small- vs. medium-sized produce growers); File S1: Survey questions.

## Abbreviations

The following abbreviations are used in this manuscript:

FSMA	Food Safety Modernization Act
PSR	Produce Safety Rule
GCFI	Gross cash flow income
CATA	Check all that apply
