# Supplementary material for: Perceptions of Produce Recall Responsibility and Compliance of FSMA Produce Safety Rule Among Small- and Medium-Sized Produce Growers
Source: Foods. 2026 Aug 21;15(16):2930. doi: 10.3390/foods15162930 (PMC13512611; doi:10.3390/foods15162930)
Supplement: Supplementary file 1 [file foods-15-02930-s001.zip › Supplementary File S1.pdf]

Supplementary Material

File SI: Study survey questions

Start of Block: IRB Number Agreement

Thank you for participating in our survey. Please answer the following questions, which will gauge your eligibility to participate in this survey.

Which of the following does your farm produce? (Please check all that apply.)

- [ ] Fruits (1)
- [ ] Vegetables (2)
- [ ] Grains (3)
- [ ] Livestock (4)
- [ ] Row crops such as field corn or soybeans (5)
- [ ] I refuse to answer. (-5555)

How would you describe the size of your farm? *Note: USDA defines small family farms as those with an annual gross cash flow income (GCFI) of less than $350,000; mid-size farms*

have a GCFI of $350,000 to $999,999; and large-scale farms are those with a GCFI of $1 million or more.

- ○ Small farm (1)
- ○ Medium-sized farm (2)
- ○ Large farm (3)
- ○ None of the above (0)
- ○ I refuse to answer. (-5555)

In which state do you currently reside?

▼ Alabama (1) ... I do not reside in the United States (-9999)

What is your gender?

- ○ Male (1)
- ○ Female (2)
- ○ Non-binary/third gender (3)
- ○ Other (4)
- ○ Prefer to self-describe (5)

____

Are you Hispanic or Latino?

- ◯ Yes (1)
- ◯ No (0)
- ◯ I refuse to answer. (-5555)

Which of the following best describes your race/ethnicity? Please check all that apply.

- ◯ Hispanic, Latino, Latinx, or Spanish (1)
- ◯ Middle Eastern or North African (2)
- ◯ White (3)
- ◯ Black or African American (4)
- ◯ Asian (5)
- ◯ American Indian or Alaska Native (6)
- ◯ Native Hawaiian or Other Pacific Islander (7)
- ◯ Some other race, ethnicity, or origin (8)
- ◯ I refuse to answer. (-5555)

Do you farm full time or part time?

- ◯ Full time (1)
- ◯ Part time (2)
- ◯ I refuse to answer. (-5555)

End of Block: Screeners

How many acres of land do you utilize to grow produce?

- 1-4.99 acres (1)
- 5-9.99 acres (2)
- 10-49.99 acres (3)
- 50-99.99 acres (4)
- 100-199.99 acres (5)
- 200-499.99 acres (6)
- 500-999.99 acres (7)
- More than 1000 acres (8)
- I refuse to answer. (-5555)

How would you describe your current business location?

- Urban (urbanized area - city or town, metropolitan area) (1)
- Suburban (outskirts of city or town, outlying area economically tied to an urban area, within commuting distance) (2)
- Rural (open and/or sparsely populated countryside, not within commuting distance to urban or suburban areas) (3)
- I refuse to answer (-5555)

Start of Block: Food Safety Questions

We will now ask about your farm's food safety specifications.

Please, read the following outbreak headline: “Multistate Outbreak of Shiga Toxin-producing *Escherichia coli* O157:H7 Infections Linked to Organic Spinach and Spring Mix Blend” *Who would you blame for this outbreak? (Select all that apply).*

- ▢ Chef (1)
- ▢ Consumer (2)
- ▢ Farmer (3)
- ▢ Government agencies (4)
- ▢ Produce packing house (5)
- ▢ Produce transportation (6)
- ▢ Retailer (7)
- ▢ ⊗All the actors (8)
- ▢ ⊗I refuse to answer. (-5555)

Please, read the following outbreak headline: **“Multistate Outbreak of *Salmonella***

Saintpaul Infections Linked to Organic Jalapeno Peppers" *Who would you blame for this outbreak? (Select all that apply).*

- ▢ Chef (1)
- ▢ Consumer (2)
- ▢ Farmer (3)
- ▢ Government agencies (4)
- ▢ Produce packing house (5)
- ▢ Produce transportation (6)
- ▢ Retailer (7)
- ▢ ⊗All the actors (8)
- ▢ ⊗I refuse to answer. (-5555)

Which of the following factors most influence your decision to comply with on farm food safety practices? (Check all that apply)

- ☐ My own belief to grow safe produce (1)
- ☐ Information available to me on safety practice (2)
- ☐ Available labor (personnel) on farm (3)
- ☐ Available utilities and time (4)
- ☐ Regulatory standards (5)
- ☐ Consumers' expectations on safe foods (6)
- ☐ Assistance from extension personnel (7)
- ☐ Other (please specify) (8) ____
- ☐ I refuse to answer. (-5555)

To what extent do you agree with the following strategies in motivating your decision to adopt on- farm food safety practices?

	Strongly disagree (1)	Disagree (2)	Neither agree nor disagree (3)	Agree (4)	Strongly agree (5)	I refuse to answer. (-5555)
Access to a centralized online platform that has all on farm food safety resources (1)	○	○	○	○	○	○
Regulatory and educational updates (2)	○	○	○	○	○	○
Tailoring regulations to small farms (3)	○	○	○	○	○	○
Liability protection (4)	○	○	○	○	○	○
Access to more trained personnel (extension and non-extension) to disseminate information (5)	○	○	○	○	○	○

Please take your time to read the information below: *The FSMA Produce Safety Rule establishes, for the first time, science-based minimum standards for the safe growing, harvesting, packing, and holding of fruits and vegetables grown for human consumption. This rule was implemented under the U.S. Food and Drug Administration (FDA). However, the rule also provides a qualified exemption and modified requirements for certain farms. To be eligible for a qualified exemption, the farm must meet two requirements:* (1) The farm must have food sales averaging less than $500,000 per year during the previous three years; and (2) The farm's sales to qualified end-users must exceed sales to all others combined during the previous three years. A qualified end-user is either (a) the consumer of the food or (b) a restaurant or retail food establishment that is located in the same state or the same Indian reservation as the farm or not more than 275 miles away. After reading the information above, do you agree that small farms should be exempt from the food safety regulation?

- ○ Yes (2)
- ○ Maybe (1)
- ○ No (0)
- ○ I refuse to answer. (-5555)

In a study conducted, about 63% consumers responded "No", which means they did not agree that qualified small size farmers be exempt from the Produce Safety Rule. Does this response from consumers surprise you?

- ○ Yes (2)
- ○ Maybe (1)
- ○ No (0)
- ○ I refuse to answer. (-5555)

To what extent do you agree with the following statements, after learning about consumers response to the exemption of qualified farms from the Produce Safety Rule.

	Strongly disagree (1)	Disagree (2)	Neither agree nor disagree (3)	Agree (4)	Strongly agree (5)	I refuse to answer. (-5555)
Consumers' expectations are different from real farm practices (1)	○	○	○	○	○	○
I am concerned that some consumers would prefer we are not exempt (2)	○	○	○	○	○	○
There is a lack of education among consumers on farm business and sales (3)	○	○	○	○	○	○
I will not be able to continue with my farming business if I am not exempt (4)	○	○	○	○	○	○

To what extent do you agree the following are **benefits** from being exempt from the FSMA Produce Safety Rule?

	Strongly disagree (1)	Disagree (2)	Neither agree nor disagree (3)	Agree (4)	Strongly agree (5)	I refuse to answer. (-5555)
Promotes direct market sales (1)	○	○	○	○	○	○
Less inspections (2)	○	○	○	○	○	○
Small farm recognition (3)	○	○	○	○	○	○
Reduced time commitment (4)	○	○	○	○	○	○
Reduced financial burden (5)	○	○	○	○	○	○

To what extent do you agree the following are **disadvantages** from being exempt from the FSMA Produce Safety Rule?

	Strongly disagree (1)	Disagree (2)	Neither agree nor disagree (3)	Agree (4)	Strongly agree (5)	I refuse to answer. (-5555)
Liability issues (1)	○	○	○	○	○	○
Reduced profits (2)	○	○	○	○	○	○
Reduced growth in expansion of farm business (3)	○	○	○	○	○	○
Discourages market reach especially with large retailers (4)	○	○	○	○	○	○

End of Block: Food Safety Questions

How would you describe your current residential location?

- Urban (urbanized area - city or town, metropolitan area) (1)
- Suburban (outskirts of city or town, outlying area economically tied to an urban area, within commuting distance) (2)
- Rural (open and/or sparsely populated countryside, not within commuting distance to urban or suburban areas) (3)
- I refuse to answer. (-5555)

Where is your household located?

- In a rural area (open and/or sparsely populated countryside) (1)
- In a town of less than 2,500 (2)
- In a town from 2,500 up to 10,000 (3)
- In a city from 10,000 up to 50,000 (4)
- From 50,000 up to 100,000 (5)
- In a city or urban area of 100,000 or more (6)
- I refuse to answer. (-5555)

Please choose the category in which the combined total **household income** fell in 2023 (yourself and any household member you live with).

- ○ Less than $25,000 (12500)
- ○ $25,000 - $49,999 (37500)
- ○ $50,000 - $74,999 (62500)
- ○ $75,000 - $99,999 (87500)
- ○ $100,000 - $149,999 (125000)
- ○ $150,000 - $199,999 (175000)
- ○ $200,000 - $299,999 (250000)
- ○ $300,000 and above (350000)
- ○ I do not know. (-9999)
- ○ I refuse to answer. (-5555)

End of Block: Household Questions

Start of Block: Owner Demographics

Now, we will ask questions about you.

What is your employment status?

- [ ] ⊗Not in the paid workforce (1)
- [ ] Full-time employment in the business (2)
- [ ] Part-time employment in the business (3)
- [ ] Full-time employment outside of the business (4)
- [ ] Part-time employment outside of the business (5)
- [ ] Employment split between the business and another outside employer (6)
- [ ] ⊗I refuse to answer. (-5555)

On average, how many hours per week do you work in your farm? (if none, choose 0)

I refuse to answer.

	0 7 14 21 28 35 42 49 56 63 70
Hours per week ()	

What is the highest grade or year of school you completed?

- ○ Did not complete high school (1)
- ○ Grade 12 or GED (High School Graduate) (2)
- ○ College 1 Year to 3 Years (Some College of Technical School) (3)
- ○ 4-year College Graduate (Example: BA, BS) (4)
- ○ Masters Degree (5)
- ○ Professional Degree beyond Bachelor's Degree (Example: MD, DDS, DVM, LLB, JD) (6)
- ○ Doctorate Degree (Example: PhD, EdD) (7)
- ○ I refuse to answer. (-5555)

What is your age in years?

▼ 18 (18) ... Prefer not to answer (-5555)

How would you describe your marital status?

- Married (1)
- Divorced (2)
- Separated (3)
- Widowed (4)
- Never married (5)
- Living with partner (6)
- I refuse to answer. (-5555)

During the year 2023, roughly what percent of household income came from your farm?

Prefer not to answer

	0 10 20 30 40 50 60 70 80 90 100
Percentage of household income that came from your farm or agribusiness ()	

End of Block: Owner Demographics
