# Supplementary material for: Perceptions of Produce Recall Responsibility and Compliance of FSMA Produce Safety Rule Among Small- and Medium-Sized Produce Growers
Source: Foods. 2026 Aug 21;15(16):2930. doi: 10.3390/foods15162930 (PMC13512611; doi:10.3390/foods15162930)
Supplement: Supplementary file 1 [file foods-15-02930-s001.zip › Updated_Supplementary tables_Produce recall responsibility.pdf]

Supplementary Figures

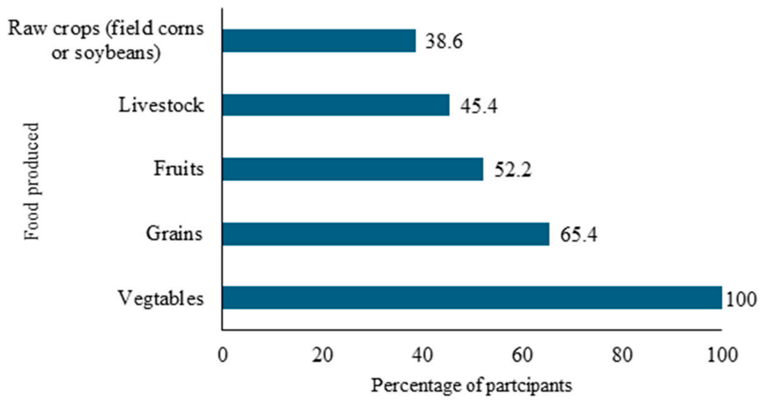

Figure S1. Distribution of foods produced by small and medium-sized produce growers (N=500)

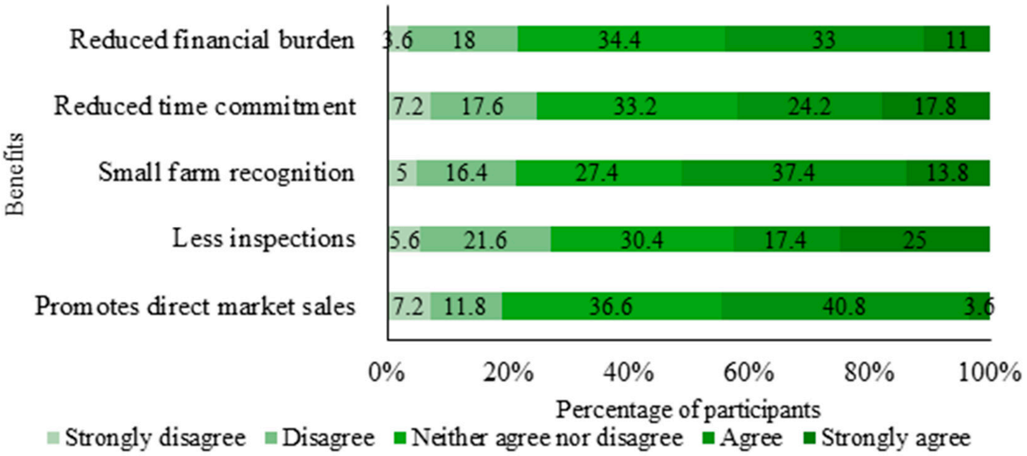

Figure S2. Produce growers' perspectives on the benefits of PSR exemptions: A Likert-scale analysis (N=500)

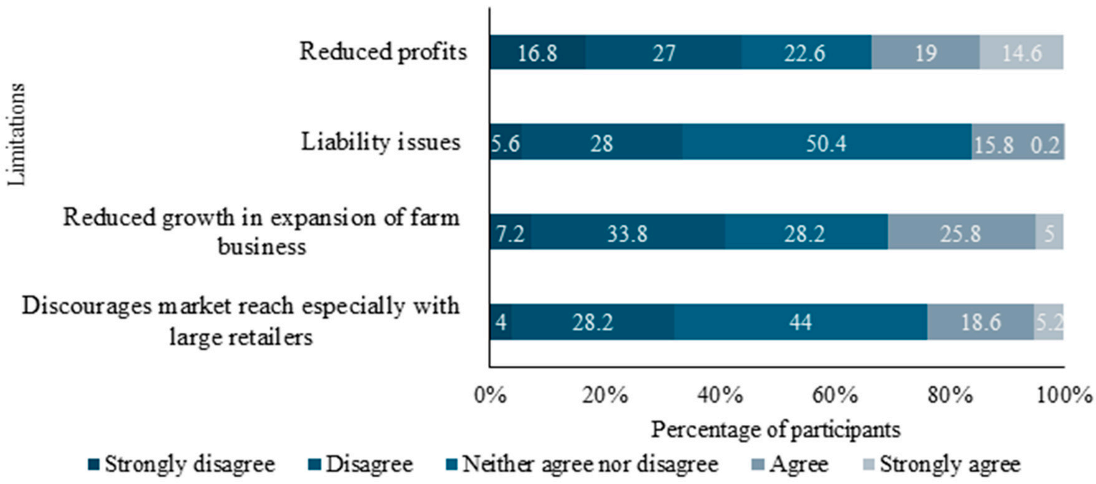

Figure S3. Produce growers' perspectives on the limitations of PSR exemptions: A Likert-scale analysis (N=500)

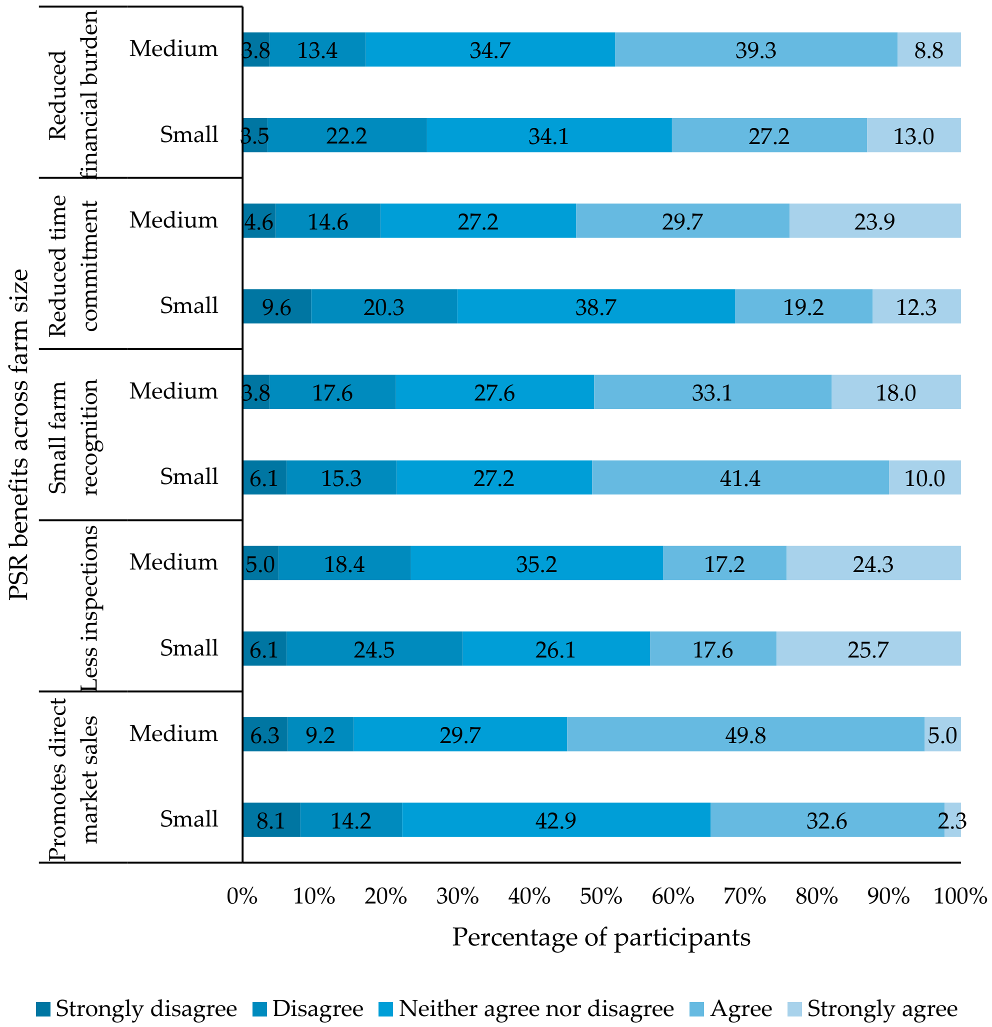

Figure S4. Frequency of responses to benefits of PSR exemption by farm size (small vs. medium-sized farms)

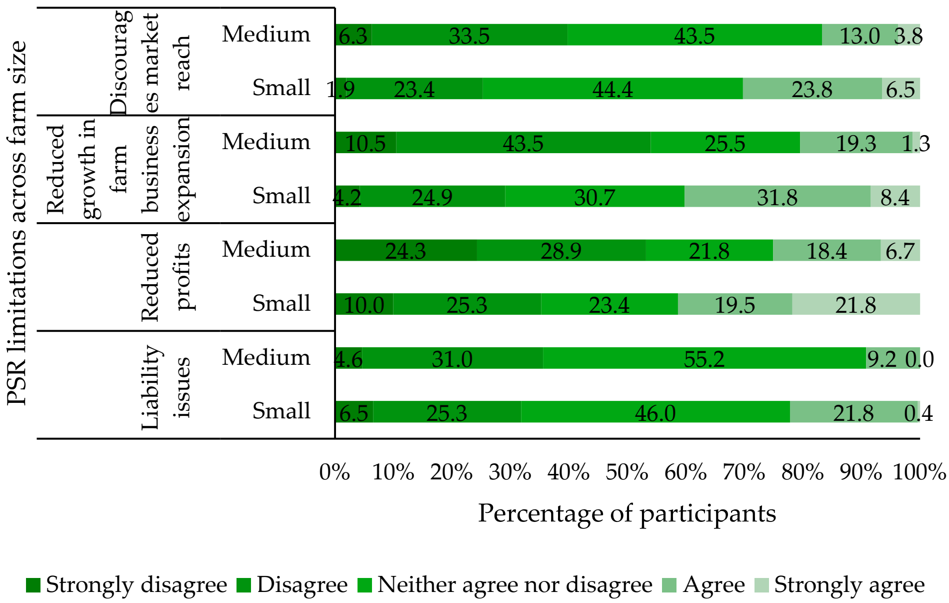

Figure S5. Frequency of responses to limitations of PSR exemptions by farm size (small vs. medium-size farms)
